# MANGO DERMATITIS

**DOI:** 10.4103/0019-5154.43215

**Published:** 2008

**Authors:** Viroj Wiwanitkit

**Affiliations:** *From Health Unit, Chulalongkorn University; Wiwanitkit House, Bangkhae, Bangkok, Thailand 10160. E-mail: wviroj@yahoo.com*

Mango, *Mangifera indica*, is a common tropical fruit. It is a favorable sweet for millions of people living in several tropical countries.^1^ Mango Dermatitis' is the common term given to allergic contact dermatitis to the sap or skin of the fruit of mango. This type of allergic reaction is rare but has become increasingly important.^2^ Here, the author reported a case of mango dermatitis. A 42-year-old female presented to the physician at a health unit with a one-day history of patchy pruritic erythema of the face, and extremities with periorbital edema. Periorbital edema was present without intraoral abnormalities or laryngeal changes. The patient denied any history of contact urticaria or new household or personal hygiene contactants. She gave the history of this illness as a common episode after ingestion of peeled mangoes. In the Health Unit, the patient was administered a single 50 mg dose of oral prednisone. She continued treatment with a five-day course of prednisone, 50 mg daily, with chlorphemiramine, 8 mg daily. Symptomatic improvement was seen over seven days. Patch testing using mango skin and mango flesh gave positive reactions. Complete avoidance of mango is suggested to this patient. Adding to the previous report of Weinstein *et al*,^1^ this is the second reported positive patch test to the mango flesh.

## References

[1] Weinstein S, Bassiri-Tehrani S, Cohen DE (2004). Allergic contact dermatitis to mango flesh. Int J Dermatol.

[2] Paschke A, Kinder H, Zunker K, Wigotzki M, Steinhart H, Wessbecher R, Vieluf I (2001). Characterization of cross-reacting allergens in mango fruit. Allergy.

